# Bioelectrical impedance parameters add incremental value to waist-to-hip ratio for prediction of metabolic dysfunction associated steatotic liver disease in youth with overweight and obesity

**DOI:** 10.3389/fendo.2024.1385002

**Published:** 2024-05-31

**Authors:** Kyungchul Song, Eun Gyung Seol, Hyejin Yang, Soyoung Jeon, Hyun Joo Shin, Hyun Wook Chae, Eun-Kyung Kim, Yu-Jin Kwon, Ji-Won Lee

**Affiliations:** ^1^ Department of Pediatrics, Yonsei University College of Medicine, Seoul, Republic of Korea; ^2^ Department of Pediatrics, Yongin Severance Hospital, Yonsei University College of Medicine, Yongin-si, Republic of Korea; ^3^ Biostatistics Collaboration Unit, Yonsei University College of Medicine, Seoul, Republic of Korea; ^4^ Department of Radiology, Research Institute of Radiological Science and Center for Clinical Imaging Data Science, Yongin Severance Hospital, Yonsei University College of Medicine, Yongin-si, Republic of Korea; ^5^ Department of Family Medicine, Yonsei University College of Medicine, Yongin Severance Hospital, Yongin-si, Republic of Korea; ^6^ Department of Family Medicine, Yonsei University College of Medicine, Severance Hospital, Seoul, Republic of Korea; ^7^ Institute for Innovation in Digital Healthcare, Yonsei University, Seoul, Republic of Korea

**Keywords:** child, adolescent, obesity, metabolic dysfunction associated steatotic liver disease, bioelectric impedance

## Abstract

**Introduction:**

Metabolic dysfunction-associated steatotic liver disease (MASLD) presents a growing health concern in pediatric populations due to its association with obesity and metabolic syndrome. Bioelectrical impedance analysis (BIA) offers a non-invasive and potentially effective alternative for identifying MASLD risk in youth with overweight or obesity. Therefore, this study aimed to assess the utility of BIA for screening for MASLD in the youth.

**Method:**

This retrospective, cross-sectional study included 206 children and adolescents aged <20 years who were overweight and obese. The correlations between anthropometric measurements and BIA parameters and alanine aminotransferase (ALT) levels were assessed using Pearson’s correlation analysis. Logistic regression analysis was performed to examine the associations between these parameters and ALT level elevation and MASLD score. Receiver operating characteristic (ROC) curves were generated to assess the predictive ability of the parameters for MASLD.

**Results:**

Pearson’s correlation analysis revealed that waist-to-hip ratio (WHR), percentage body fat (PBF), and BIA parameters combined with anthropometric measurements were correlated with ALT level. Logistic regression revealed that WHR, skeletal muscle mass/WHR, PBF-WHR, fat-free mass/WHR, and appendicular skeletal muscle mass/WHR were correlated with ALT level elevation after adjusting for age, sex, and puberty. WHR, PBF-WHR, and visceral fat area (VFA)-WHR were positively correlated with the MASLD score in the total population after adjusting for age, sex, and puberty. PBF-WHR and VFA-WHR were correlated with the MASLD score even in youth with a normal ALT level. The cutoff points and area under the ROC curves were 34.6 and 0.69 for PBF-WHR, respectively, and 86.6 and 0.79 for VFA-WHR, respectively.

**Discussion:**

This study highlights the utility of combining BIA parameters and WHR in identifying the risk of MASLD in overweight and obese youth, even in those with a normal ALT level. BIA-based screening offers a less burdensome and more efficient alternative to conventional MASLD screening methods, facilitating early detection and intervention in youth at risk of MASLD.

## 1 Introduction

Nonalcoholic fatty liver disease (NAFLD) is a chronic liver condition characterized by the accumulation of excess fat in the liver, encompassing a spectrum from simple steatosis to nonalcoholic steatohepatitis and hepatic fibrosis (1, 2). NAFLD has been associated with cardiovascular and metabolic risk factors as well as the development of hepatic cirrhosis and hepatic cancer, even in children and adolescents (1, 3, 4). A meta-analysis revealed a global increase in the prevalence of NAFLD from 4.62% in 2000 to 9.02% in 2017 among children and adolescents (5). Notably, in Korea, the prevalence of NAFLD increased from 45.8% to 62.5% during the outbreak of coronavirus disease 2019 among children and adolescents with obesity (6).

Early screening for NAFLD is crucial for prevention of severe complications, given that NAFLD is often asymptomatic and is reversible with appropriate management during the initial stages (1, 4). Therefore, the Endocrine Society recommends alanine transaminase (ALT) screening for early detection of NAFLD in children with overweight and obesity (7). The North American Society of Pediatric Gastroenterology, Hepatology, and Nutrition recommends imaging studies, including ultrasonography, for NAFLD screening in children with abnormal ALT levels (8).

However, the necessity for blood sampling for ALT screening creates a potential burden on children. Furthermore, some children diagnosed with NAFLD may exhibit normal ALT levels, which can be elevated in various liver-related conditions (1). Consequently, initiatives have been undertaken to explore biomarkers to compensate for the limitations of ALT screening or imaging studies (1, 9). Despite these efforts, additional research in pediatric populations is warranted.

While body mass index (BMI) serves as a diagnostic tool for obesity, individuals whose BMI falls within the normal range may still present a metabolically unhealthy profile commonly associated with obesity; conversely, some individuals who are overweight or obese may be metabolically healthy (10). According to a recent study on the Korean population, the prevalence of metabolically healthy adolescents with overweight or obesity has remained steady, while the number of adolescents with overweight or obesity increased (11). Identifying metabolically healthy individuals with overweight is encumbered by the inherent limitations of BMI measurements, as they lack the ability to distinguish between body fat and muscle mass (12). A meta-analysis reported that bioelectrical impedance analysis (BIA) is a practical method for evaluating body fat in children and adolescents (13). However, investigations on the associations of BIA parameters with pediatric fatty liver disease are limited.

Recently, international experts have released a consensus statement regarding the renaming of fatty liver disease to metabolic dysfunction-associated steatotic liver disease (MASLD) (14). This new terminology reflects the association this condition with hepatic steatosis and the inclusion of at least one out of five cardiometabolic risk factors, which are associated with elements of metabolic syndrome. While this new concept enables the assessment of MASLD as a cardiometabolic risk factor by emphasizing the close pathophysiological link between fatty liver and metabolic dysfunction and insulin resistance, investigations on MASLD in pediatrics are limited. Considering adverse trends in cardiovascular risk factors, including metabolic syndrome, screening strategies for MASLD in pediatric patients are needed (2, 15).

In this study, we aimed to investigate the utility of BIA in identifying different metabolic profiles among individuals who are overweight and obese, providing valuable insights into more effective and less burdensome screening methods for MASLD in the pediatric population. Therefore, our study focused on the development of MASLD-related parameters using anthropometric measurements and BIA parameters that are specifically tailored for children and adolescents who are overweight and obese. Considering the metabolic diversity within this population, our objectives were to (1) examine the associations of BIA parameters with ALT level and MASLD, (2) compare the associations between MASLD and traditional anthropometric data and BIA parameters, and (3) establish valid cutoff values for BIA-related parameters for the prediction of MASLD.

## 2 Methods

### 2.1 Study population

This retrospective, cross-sectional study investigated participants aged <20 years who visited the Department of Pediatrics or Department of Family Medicine at Yongin Severance Hospital with a chief complaint of overweight or obesity from March 2020 to July 2023. Among them, 208 participants who underwent BIA were enrolled. Among these participants, those with missing ALT data (n=2) were excluded. None of the participants were alcohol drinkers or had hepatitis B virus and hepatitis C virus infection. Finally, 206 participants (107 boys and 99 girls) were enrolled.

### 2.2 Anthropometric measurements and laboratory analyses

Height measurements were acquired with a precision of 0.1 cm, whereas body weight was measured using an electronic scale accurate to 0.01 kg. BMI was computed by dividing weight in kilograms by the square of the height in meters (kg/m^2^). Height, weight, and BMI were expressed with respect to the standard deviation scores (SDSs) established by the 2017 Korean National Growth Charts (16). Waist circumference (WC) was measured by placing a measuring tape horizontally at the midpoint between the lowest rib and the iliac crest. Hip circumference was measured around the widest point of the hips, i.e., around the buttocks.

The waist-to-height ratio (WHtR) was calculated by dividing WC by height, while the waist-to-hip ratio (WHR) was calculated by dividing WC by hip circumference. Obesity was defined as BMI above the 95th percentile, and overweight was defined as a BMI between the 85th percentile and the 95th percentile according to the 2017 Korean National Growth Charts (16, 17). Participants with normal BMI, BMI below the 85th percentile, were not included in this study. Puberty was identified by any pubertal development corresponding to a Tanner stage of ≥2 (18). Blood samples were collected from the antecubital vein after an 8-h fast and were then processed and immediately refrigerated. The serum levels of aspartate transaminase and ALT were measured using an absorbance assay with a Roche Cobas 8000 c702 (Roche Diagnostics, Mannheim, Germany). The concentrations of hepatitis B surface antigen and anti-HCV antibodies were measured using a Roche Cobas 8000 c702 (Roche Diagnostics, Mannheim, Germany).

### 2.3 BIA and BIA markers combined with WHR

Skeletal muscle mass (SMM), fat-free mass (FFM), appendicular skeletal muscle mass (ASM), percentage body fat (PBF), WHtR, WHR, and visceral fat area (VFA) were assessed using the InBody720 body composition analyzer (Biospace, Seoul, South Korea). ALT level elevation was defined as ALT level >26 IU/L for male participants and >22 IU/L for female participants, excluding individuals with hepatitis B or C viral infection and alcohol drinkers (6, 19).

We established the following BIA parameters: 1) SMM divided by BMI SDS (SMM/BMI SDS), SMM divided by WHtR (SMM/WHtR), and SMM divided by WHR (SMM/WHR). FFM and ASM were calculated in the same manner: 2) PBF × BMI SDS (PBF-BMI SDS), PBF × WC (PBF-WC), PBF ×WHtR (PBF-WHtR), and PBF × WHR (PBF-WHR). The VFA was calculated in the same manner.

### 2.4 Diagnosis of MASLD using abdominal ultrasonography

The diagnosis of steatotic liver disease was based on abdominal ultrasonography performed with a C1–8 MHz convex transducer of the Aplio i800 (Canon Medical Systems, Otawara, Japan) and C1–6 MHz of the LOGIQ E10 (GE Healthcare, Wauwatosa, WI, USA) by a pediatric radiologist with 14 years of experience. Participants were categorized into four groups based on the presence and severity of steatotic liver disease determined by assessing the degree of liver tissue echogenicity, the level of contrast between the liver and right kidney, and the visibility of vascular structures (1, 20). A hepatic fat accumulation grade of 1–3 was considered indicative of steatotic liver disease, while a hepatic fat accumulation grade of 0 indicated normal conditions.

According to international consensus, MASLD is defined as a steatotic liver disease with the presence of at least one of five cardiometabolic risk factors (14). Because all the participants in our study were overweight or obese, we defined steatotic liver disease as MASLD.

### 2.5 Statistical analyses

All continuous variables are presented as means ± standard deviations, while categorical variables are presented as numbers (percentages). Continuous variables were compared using the independent t test, while categorical variables were compared using the chi-squared test. Pearson’s correlation was employed to determine the associations between the BIA parameters and the WHR and ALT. Logistic regression analysis was performed to determine the associations of WHR alone and WHR combined with BIA parameters with ALT elevation and MASLD. The area under the receiver operating characteristic (ROC) curve (AUC) was computed to assess the diagnostic efficacy of these parameters for identifying MASLD. The data were analyzed using R, version 4.2.2 (The R Foundation for Statistical Computing, Vienna, Austria; http://www.R-project.org, accessed on October 31, 2022). Statistical significance was set at p values ≤0.05.

### 2.6 Ethics statement

This study adhered to the ethical guidelines outlined in the 1975 Declaration of Helsinki and was approved by the Institutional Review Board of Yonsei University Yongin Severance Hospital (IRB number: 9–2023-0071); the requirement for informed consent was waived.

## 3 Results

### 3.1 Participants’ clinical characteristics

**Table 1** presents the clinical characteristics of the study population with respect to sex and pubertal status. The proportion of puberty and obesity was greater in girls than in boys. In boys, the WC, WHtR, WHR, SMM, SMM/WC, SMM/WHtR, SMM/WHR, FFM, FFM/WC, FFM/WHtR, FFM/WHR, ASM, ASM/WC, ASM/WHtR, ASM/WHR, VFA, VFA-BMI SDS, VFA-WC, VFA-WHtR, VFA-WHR, and ALT level were greater, while PBF was lower than that in girls. The prevalence of obesity was greater in the pubertal group than in the prepubertal group. WHR, SMM, SMM/BMI SDS, SMM/WC, SMM/WHtR, SMM/WHR, FFM, FFM/BMI SDS, FFM/WC, FFM/WHtR, FFM/WHR, ASM, ASM/BMI-SDS, ASM/WC, ASM/WHtR, and ASM/WHR were greater in the puberty group than in the prepubertal group. In contrast, WHR, PBF, PBF-WHtR and PBF-WHR were lower in the pubertal group than in the prepubertal group.

**Table 1:** Participants’ baseline characteristics with respect to sex and pubertal status.

	Total (n=206)	Boys (n=107)	Girls (n=99)	*p*	Prepuberty (n=26)	Puberty (n=180)	*p*
Age		12.0 ± 2.7	11.6 ± 3.3	0.268	10.2 ± 1.2	12.1 ± 3.1	<0.001
Male, n (%)	107 (51.94)	–	–		21 (80.77)	86 (47.78)	0.002
Puberty, n (%)	180 (87.38)	86 (80.37)	94 (94.95)	0.002	–	–	
Height, cm	153.4 ± 12.7	156.4 ± 15.6	150.0 ± 15.2	0.003	144.7 ± 11.1	154.7 ± 15.9	<0.001
Height SDS	1.05 ± 1.24	1.01 ± 1.15	1.08 ± 1.33	0.710	0.80 ± 1.25	1.08 ± 1.24	0.276
Weight, kg	67.5 ± 22.9	72.0 ± 24.9	62.6 ± 19.4	0.003	58.0 ± 17.5	68.9 ± 23.3	0.024
Weight SDS	2.53 ± 1.06	2.53 ± 1.21	2.53 ± 0.85	0.964	2.28 ± 1.14	2.56 ± 1.04	0.205
BMI	27.82 ± 4.96	28.49 ± 5.21	27.10 ± 4.58	0.044	27.10 ± 4.73	27.92 ± 5.00	0.428
BMI SDS	2.71 ± 1.03	2.71 ± 1.14	2.72 ± 0.89	0.959	2.67 ± 1.03	2.72 ± 1.03	0.833
Obesity, n (%)	181 (92.35)	91 (88.4)	90 (96.8)	0.027	20 (76.9)	161 (94.7)	0.007
WC	87.91 ± 12.10	91.27 ± 12.40	84.35 ± 10.76	<0.001	87.67 ± 13.85	87.95 ± 11.84	0.916
WHtR	0.58 ± 0.06	0.59 ± 0.07	0.57 ± 0.06	0.009	0.60 ± 0.07	0.58 ± 0.06	0.143
WHR	0.92 ± 0.05	0.94 ± 0.05	0.90 ± 0.05	<0.001	0.94 ± 0.05	0.92 ± 0.05	0.028
SMM	22.38 ± 8.28	24.48 ± 9.17	20.12 ± 6.51	<0.001	18.03 ± 4.97	23.01 ± 8.48	<0.001
SMM/BMI SDS	9.72 ± 8.34	10.62 ± 8.02	8.73 ± 8.62	0.109	7.43 ± 2.57	10.06 ± 8.84	0.002
SMM/WC	0.24 ± 0.06	0.25 ± 0.06	0.22 ± 0.06	0.023	0.20 ± 0.03	0.24 ± 0.06	<0.001
SMM/WHtR	38.39 ± 13.17	41.10 ± 13.95	35.46 ± 11.66	0.002	29.89 ± 6.33	39.62 ± 13.46	<0.001
SMM/WHR	23.11 ± 8.36	24.45 ± 8.87	21.65 ± 7.55	0.029	19.15 ± 4.81	23.79 ± 8.66	<0.001
PBF	38.72 ± 5.90	37.79 ± 6.10	39.73 ± 5.53	0.018	40.85 ± 5.07	38.41 ± 5.96	0.049
PBF-BMI SDS	108.70 ± 53.29	106.34 ± 56.72	111.30 ± 49.38	0.510	113.64 ± 54.64	107.97 ± 53.20	0.614
PBF-WC	3404.16 ± 797.22	3461.91 ± 829.72	3343.03 ± 761.42	0.326	3630.73 ± 929.26	3366.40 ± 770.11	0.125
PBF-WHtR	38.39 ± 13.17	22.69 ± 5.78	22.84 ± 5.10	0.848	24.75 ± 5.54	22.48 ± 5.39	0.047
PBF-WHR	23.11 ± 8.36	35.61 ± 6.80	35.30 ± 5.66	0.747	38.61 ± 6.35	34.91 ± 6.11	0.006
FFM	41.14 ± 13.94	44.60 ± 15.42	37.39 ± 11.06	<0.001	33.82 ± 8.48	42.19 ± 14.27	<0.001
FFM/BMI SDS	18.01 ± 15.82	19.48 ± 14.76	16.39 ± 16.84	0.166	14.02 ± 4.86	18.60 ± 16.77	0.004
FFM/WC	0.44 ± 0.10	0.45 ± 0.10	0.42 ± 0.09	0.040	0.38 ± 0.05	0.44 ± 0.10	<0.001
FFM/WHtR	70.64 ± 22.20	74.99 ± 23.42	65.94 ± 19.87	0.003	56.15 ± 10.58	72.73 ± 22.67	<0.001
FFM/WHR	42.68 ± 14.15	44.80 ± 14.95	40.37 ± 12.93	0.042	35.95 ± 8.14	43.85 ± 14.66	<0.001
ASM	16.32 ± 6.46	17.90 ± 7.00	14.62 ± 5.36	<0.001	12.76 ± 3.66	16.83 ± 6.62	<0.001
ASM/BMI SDS	7.05 ± 5.88	7.77 ± 5.92	6.26 ± 5.76	0.068	5.24 ± 1.76	7.32 ± 6.22	<0.001
ASM/WC	0.17 ± 0.05	0.18 ± 0.05	0.16 ± 0.05	0.025	0.14 ± 0.02	0.17 ± 0.05	<0.001
ASM/WHtR	28.01 ± 10.49	30.07 ± 10.84	25.79 ± 9.67	0.003	21.16 ± 4.72	29.00 ± 10.73	<0.001
ASM/WHR	16.78 ± 6.59	17.82 ± 6.79	15.66 ± 6.20	0.034	13.58 ± 3.57	17.34 ± 6.83	<0.001
VFA	119.59 ± 46.99	129.52 ± 50.40	108.86 ± 40.59	0.001	117.23 ± 48.53	119.93 ± 46.89	0.785
VFA-BMI SDS	358.84 ± 275.67	396.63 ± 327.34	317.12 ± 197.50	0.036	355.90 ± 266.81	359.27 ± 277.70	0.954
VFA-WC	10418.62 ± 5222.15	11536.5 ± 5603.2	9235.0 ± 4524.2	0.003	10919.5 ± 6186.3	10335.1 ± 5062.9	0.606
VFA-WHtR	72.04 ± 36.05	79.78 ± 40.32	63.68 ± 28.72	0.001	73.06 ± 37.45	71.89 ± 35.95	0.878
VFA-WHR	105.32 ± 43.45	115.58 ± 44.68	94.17 ± 39.39	0.001	112.37 ± 51.55	104.09 ± 41.98	0.381
AST	31.91 ± 25.57	34.18 ± 28.26	29.42 ± 22.15	0.183	39.60 ± 38.84	30.81 ± 23.04	0.280
ALT	39.26 ± 44.51	46.15 ± 53.06	31.72 ± 31.29	0.019	53.88 ± 71.07	37.18 ± 39.20	0.261
ALT level elevation	106 (52.74)	60 (57.1)	46 (47.9)	0.191	15 (60.0)	91 (51.7)	0.437
MASLD, n (%)	62 (67.39)	33 (70.2)	29 (64.4)	0.555	8 (66.7)	54 (67.5)	>0.999

The values are presented as the mean ± standard deviation (standard error) and categorical data as numbers (%). SDS, standard deviation score; BMI, body mass index; WC, waist circumference; WHtR, waist-to-height ratio; WHR, waist-to-hip ratio; SMM, skeletal muscle mass; PBF, percentage body fat; FFM, fat free mass; ASM, appendicular skeletal mass; VFA, visceral fat area; AST, aspartate transaminase; ALT, alanine transaminase; MASLD, metabolic dysfunction associated liver disease.

### 3.2 Correlation of anthropometric data and BIA parameters with ALT level

WHR was the only anthropometric factor correlated with ALT in the total population (r=0.25, p=0.001), boys (p=0.030), the prepubertal group (p=0.041), and the pubertal group (p=0.024) (**Table 2**). Among the BIA parameters, ALT level was significantly correlated with SMM/WC, PBF, PBF-WHtR, PBF-WHR, and FFM/WC in boys and with PBF and the PBF-WHR in girls. No significant correlation was detected between these parameters and ALT level in the prepubertal group. In the pubertal group, ALT level exhibited significant correlations with the PBF, PBF-WC, PBF-WHtR, and PBF-WHR.

**Table 2:** Correlation of anthropometric data and BIA parameters with ALT.

	Total		Boys		Girls		Prepuberty		Puberty	
	r	*p*	r	*p*	r	*p*	r	*p*	r	*p*
BMI SDS	0.07	0.355	0.08	0.447	0.05	0.602	0.10	0.63	0.06	0.435
WC	0.01	0.936	-0.07	0.544	0.01	0.908	-0.02	0.935	0.01	0.900
WHtR	0.10	0.142	0.08	0.407	0.07	0.494	0.16	0.456	0.08	0.316
WHR	0.25	0.001	0.23	0.030	0.20	0.082	0.42	0.041	0.19	0.024
SMM	-0.09	0.223	-0.13	0.197	-0.16	0.115	-0.18	0.388	-0.05	0.473
SMM/BMI SDS	-0.01	0.865	-0.04	0.667	-0.01	0.898	-0.18	0.394	0.01	0.854
SMM/WC	-0.18	0.017	-0.22	0.035	-0.20	0.067	-0.31	0.148	-0.16	0.052
SMM/WHtR	-0.12	0.081	-0.16	0.095	-0.18	0.086	-0.25	0.23	-0.09	0.231
SMM/WHR	-0.15	0.046	-0.20	0.058	-0.16	0.167	-0.25	0.238	-0.13	0.115
PBF	0.21	0.003	0.27	0.005	0.19	0.060	0.29	0.167	0.19	0.013
PBF-BMI SDS	0.11	0.109	0.13	0.172	0.11	0.303	0.15	0.464	0.10	0.186
PBF-WC	0.17	0.030	0.16	0.136	0.16	0.155	0.11	0.606	0.17	0.042
PBF-WHtR	0.18	0.011	0.21	0.033	0.14	0.165	0.25	0.238	0.15	0.049
PBF-WHR	0.32	<0.001	0.35	<0.001	0.29	0.010	0.38	0.064	0.30	<0.001
FFM	-0.09	0.227	-0.13	0.199	-0.16	0.127	-0.18	0.400	-0.05	0.477
FFM/BMI SDS	-0.01	0.910	-0.04	0.709	-0.01	0.956	-0.17	0.407	0.02	0.819
FFM/WC	-0.18	0.016	-0.22	0.037	-0.20	0.068	-0.32	0.131	-0.16	0.052
FFM/WHtR	-0.13	0.075	-0.17	0.091	-0.17	0.092	-0.26	0.217	-0.09	0.223
FFM/WHR	-0.15	0.046	-0.20	0.059	-0.15	0.179	-0.26	0.226	-0.13	0.12
ASM	-0.08	0.242	-0.12	0.228	-0.16	0.116	-0.16	0.437	-0.05	0.496
ASM/BMI SDS	-0.01	0.837	-0.04	0.666	-0.03	0.809	-0.18	0.401	0.01	0.871
ASM/WC	-0.16	0.032	-0.20)	0.062	-0.20	0.072	-0.27	0.21	-0.14	0.083
ASM/WHtR	-0.12	0.101	-0.15	0.125	-0.17	0.092	-0.22	0.293	-0.09	0.262
ASM/WHR	-0.14	0.064	-0.19	0.082	-0.16	0.168	-0.23	0.293	-0.12	0.148
VFA	0.10	0.151	0.06	0.552	0.09	0.379	0.15	0.476	0.09	0.217
VFA-BMI SDS	0.08	0.269	0.05	0.618	0.09	0.407	0.09	0.677	0.08	0.302
VFA-WC	0.07	0.350	0.01	0.956	0.12	0.297	0.07	0.748	0.06	0.437
VFA-WHR	0.16	0.044	0.10	0.363	0.18	0.107	0.19	0.387	0.13	0.108
VFA-WHtR	0.10	0.159	0.06	0.547	0.08	0.422	0.14	0.521	0.09	0.236

BIA, bioelectrical impedance analysis; ALT, alanine transaminase; CI, confidence interval; BMI, body mass index; SDS, standard deviation score; WC, waist circumference; WHtR, waist-to-height ratio; WHR, waist-to-hip ratio; SMM, skeletal muscle mass; PBF, percentage body fat; FFM, fat free mass; ASM, appendicular skeletal mass; VFA, visceral fat area.

### 3.3 Logistic regression analysis for ALT elevation

Considering the significance of WHR among the anthropometric variables, logistic regression analysis was performed to determine its association with ALT elevation by selecting indicators that integrated WHR into the representative BIA parameters after adjusting for age, sex (if applicable) and puberty (if applicable) (**Table 3**). In the total population, WHR (per 100) (odds ratio [OR], [95% confidence interval (CI)]=1.09 [1.02–1.17], p=0.017), SMM/WHR, PBF-WHR, FMM/WHR, and ASM/WHR were significantly associated with ALT level elevation after adjusting for age, sex, and puberty. According to the sex-specific analysis, ALT level elevation was significantly associated with PBF-WHR (OR [95% CI]=1.13 [1.04–1.23], p=0.005) and VFA-WHR (OR [95% CI]=1.01 [1.00–1.02], p=0.045) in boys and with SMM/WHR (OR [95% CI]=0.83 [0.73–0.94], p=0.004), FFM/WHR (OR [95% CI]=0.89 [0.83–0.96], p=0.003), and ASM/WHR (OR [95% CI]=0.81 [0.70–0.94], p=0.005) in girls after adjusting for age and puberty. In the pubertal group, significant associations were detected between WHR (per 100) (OR [95% CI]=1.09 [1.01–1.18], p=0.024), SMM/WHR (OR [95% CI]=0.92 [0.85–0.99], p=0.021), PBF-WHR (OR [95% CI]=1.10 [1.04–1.17], p=0.002), FFM/WHR (OR [95% CI]=0.95 [0.91–0.99], p=0.018), ASM/WHR (OR [95% CI]=0.89 [0.81–0.98], p=0.019), and VFA-WHR (OR [95% CI]=1.01 [1.00–1.02], p=0.039), and ALT level elevation after adjusting for age and sex, while no such significant associations were detected in the prepubertal group.

**Table 3:** Logistic regression analysis for ALT level elevation†.

	Total		Boys			Girls		Prepuberty		Puberty	
	OR (95% CI)	p*	OR (95% CI)		p**	OR (95% CI)	p **	OR (95% CI)	p ***	OR (95% CI)	p***
WHR (per 100)	1.09 (1.02–1.17)	0.017	1.08 (0.98–1.20)		0.131	1.09 (0.99–1.20)	0.085	1.07 (0.87–1.33)	0.505	1.09 (1.01–1.18)	0.024
SMM/WHR	0.91 (0.85–0.98)	0.013	1.00 (0.90–1.10)		0.921	0.83 (0.73–0.94)	0.004	0.89 (0.68–1.17)	0.409	0.92 (0.85–0.99)	0.021
PBF-WHR	1.09 (1.03–1.15)	0.003	1.13 (1.04–1.23)		0.005	1.04 (0.96–1.13)	0.334	1.01 (0.87–1.17)	0.909	1.10 (1.04–1.17)	0.002
FMM/WHR	0.95 (0.91–0.99)	0.011	1.00 (0.94–1.05)		0.888	0.89 (0.83–0.96)	0.003	0.93 (0.79–1.10)	0.385	0.95 (0.91–0.99)	0.018
ASM/WHR	0.89 (0.82–0.98)	0.013	0.99 (0.87–1.12)		0.892	0.81 (0.70–0.94)	0.005	0.88 (0.62–1.26)	0.484	0.89 (0.81–0.98)	0.019
VFA-WHR	1.01 (1.00–1.02)	0.079	1.01 (1.00–1.02)		0.045	1.00 (0.99–1.01)	0.724	1.00 (0.98–1.02)	0.708	1.01 (1.00–1.02)	0.039

*Adjusted for age, sex, and puberty.

**Adjusted for age and puberty.

***Adjusted for age and sex.

†ALT level elevation was defined as ALT level >26 IU/L for male participants and >22 IU/L for female participants.

ALT, alanine transaminase; OR, odds ratio; CI, confidence interval; WHR, waist-to-hip ratio; SMM, skeletal muscle mass; PBF, percentage body fat; FFM, fat free mass; ASM, appendicular skeletal mass; VFA, visceral fat area; BMI, body mass index; SDS, standard deviation score.

### 3.4 Logistic regression of WHR and BIA parameters for MASLD

**Figure 1:**
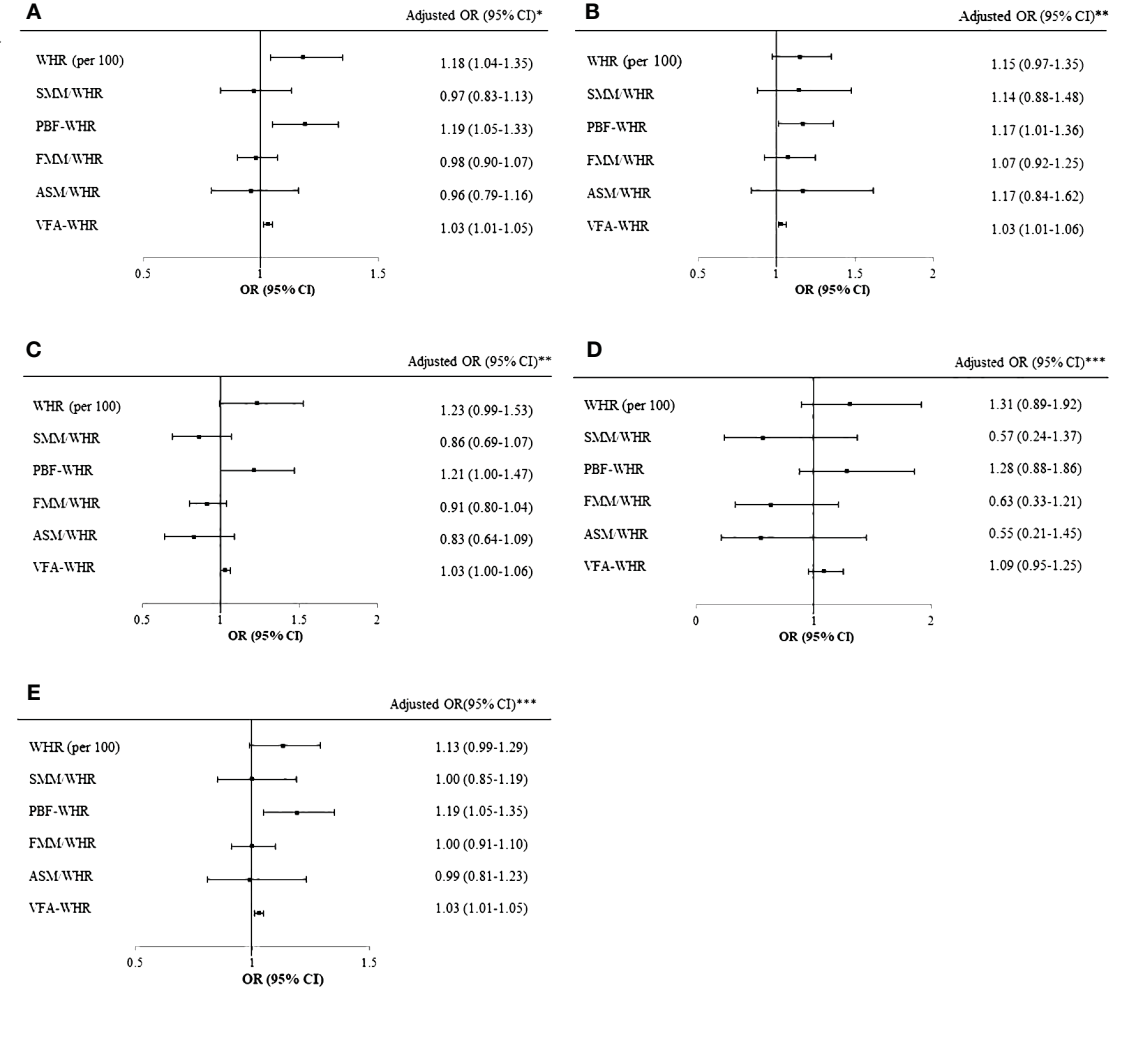
Logistic regression of WHR and BIA parameters for MASLD. **(A)** Logistic regression of WHR and BIA parameters for MASLD in the total cohort after adjusting for age, sex, and puberty. **(B)** Logistic regression of WHR and BIA parameters for MASLD in boys after adjusting for age and puberty. **(C)** Logistic regression of WHR and BIA parameters for MASLD in girls after adjusting for age and puberty. **(D)** Logistic regression of WHR and BIA parameters for MASLD in the prepubertal group after adjusting for age and sex. **(E)** Logistic regression of WHR and BIA parameters for MASLD in the pubertal group after adjusting for age and sex. *Adjusted for age, sex, and puberty. **Adjusted for age and puberty. ***Adjusted for age and sex. WHR, waist-to-hip ratio; BIA, bioelectrical impedance analysis; MASLD, metabolic dysfunction associated steatotic liver disease; SMM, skeletal muscle mass; PBF, percentage of body fat; FFM, fat-free mass; ASM, appendicular skeletal mass; VFA, visceral fat area; BMI, body mass index; SDS, standard deviation score.

**Figure 1** shows the logistic regression analysis results for WHR and WHR combined with BIA parameters for the diagnosis of MASLD by abdominal ultrasonography in the total population (**Figure 1A**), boys (**Figure 1B**), girls (**Figure 1C**), prepubertal group (**Figure 1D**), and pubertal group (**Figure 1E**). In the overall population, the WHR (per 100; OR [95% CI]=1.18 [1.04–1.35]), PBF-WHR (OR [95% CI]=1.19 [1.05–1.33]), and VFA-WHR (OR [95% CI]=1.03 [1.01–1.05]) showed significant associations with MASLD after adjusting for age, sex, and puberty. The ORs (95% CIs) were 1.17 (1.01–1.36) for PBF-WHR and 1.03 (1.01–1.06) for VFA-WHR in boys and 1.21 (1.00–1.47) for PBF-WHR and 1.03 (1.00–1.06) for VFA-WHR in girls. In the prepubertal group, the combined WHR and BIA parameters did not exhibit any significant association with MASLD. However, in the pubertal group, MASLD was significantly associated with the PBF-WHR (OR [95% CI]=1.19 [1.05–1.35]) and VFA-WHR (OR [95% CI]=1.03 [1.01–1.05]).

**Figure 2:**
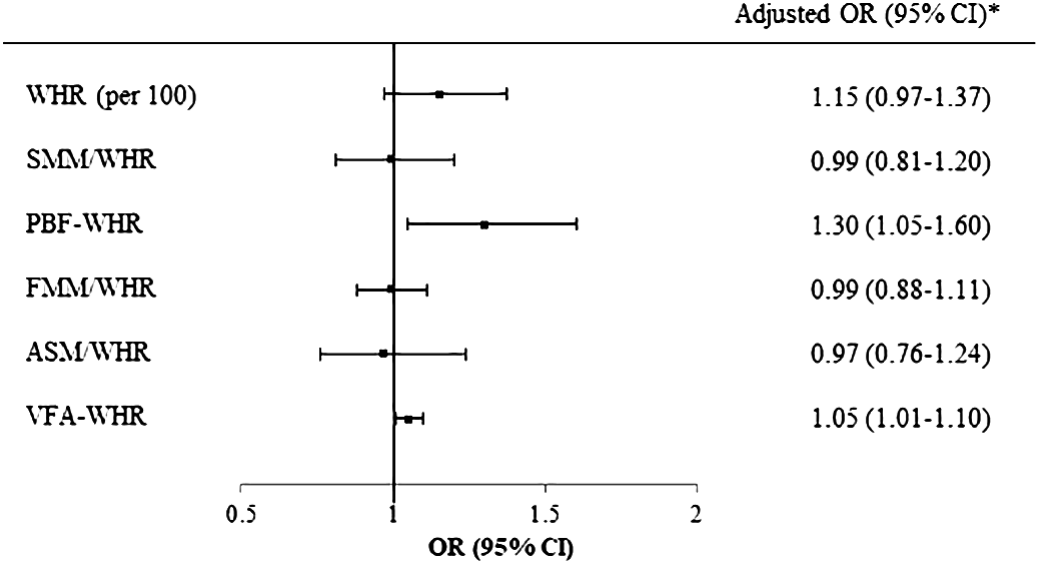
Logistic regression of WHR and BIA parameters for MASLD in participants with normal ALT. *Adjusted for age, sex, and puberty. WHR, waist-to-hip ratio; BIA, bioelectrical impedance analysis; MASLD, metabolic dysfunction-associated steatotic liver disease; ALT, alanine aminotransferase; SMM, skeletal muscle mass; PBF, percentage of body fat; FFM, fat-free mass; ASM, appendicular skeletal mass; VFA, visceral fat area.

Logistic regression analysis was performed to investigate the associations of the MASLD with WHR and combined parameters in individuals with normal ALT levels. The analysis revealed that PBF-WHR (OR [95% CI]=1.30 [1.05–1.60]) and VFA-WHR (OR [95% CI]=1.05 [1.01–1.10]) were significantly associated with MASLD (**Figure 2**).

### 3.5 Cutoff values and AUCs for each variable for predicting MASLD

**Figure 3:**
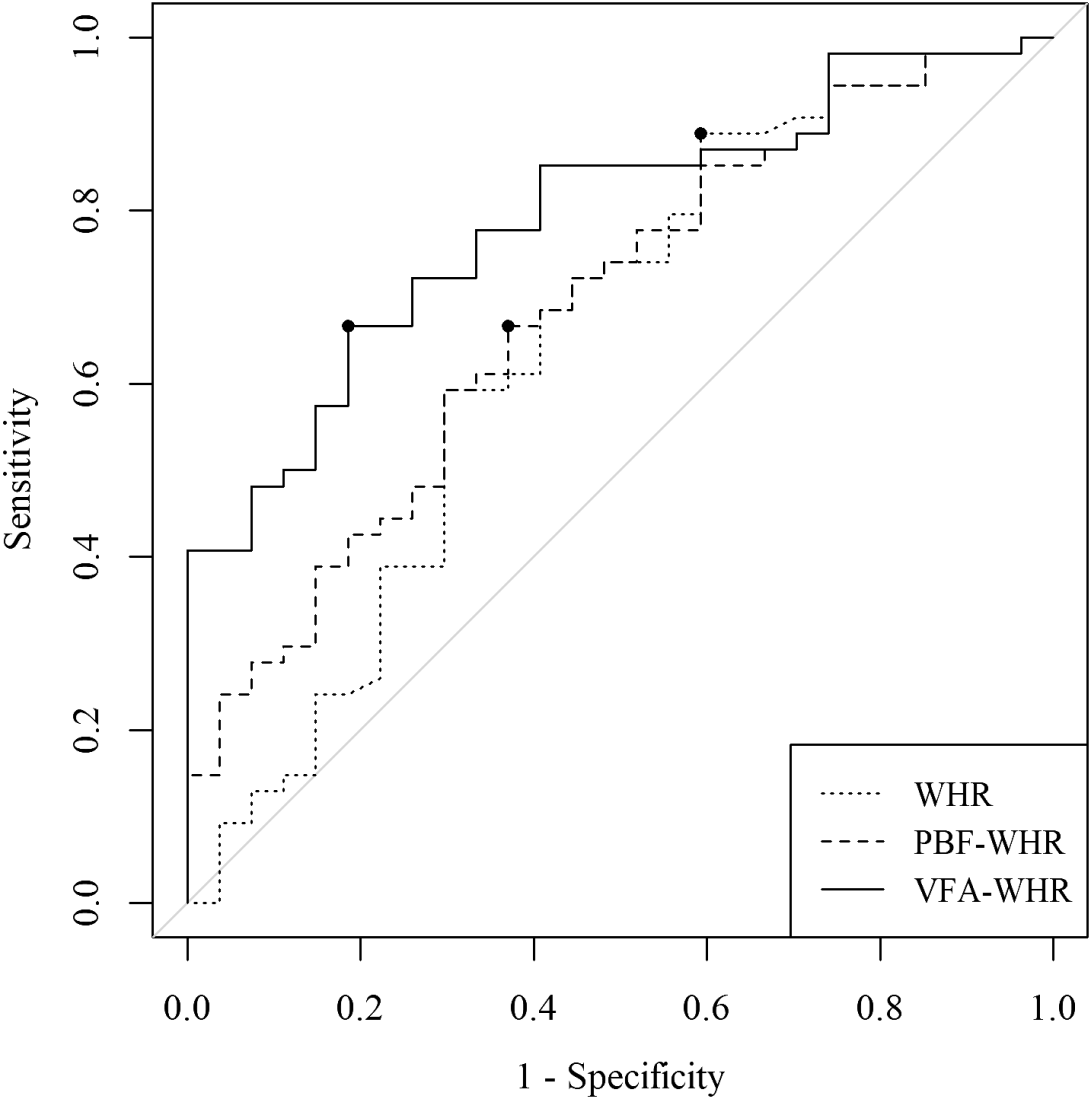
Comparison of the AUC and the corresponding cutoff points for WHR, PBF-WHR, and VFA-WHR. The dots on the curves represent the cutoff points for WHR, PBF-WHR, and VFA-WHR. AUC, area under the receiver operating characteristic curve; WHR, waist-to-hip ratio; SMM, skeletal muscle mass; PBF, percentage of body fat; VFA, visceral fat areaTables.

**Figure 3** displays the ROC curve along with the corresponding cutoff points for WHR, PBF-WHR, and VFA-WHR in the total population. The AUC (95% CI) and associated cutoff points were 0.65 (0.51–0.78) and 0.88 for WHR, 0.69 (0.56–0.81) and 34.6 for PBF-WHR, and 0.79 (0.69–0.89) and 86.6 for VFA-WHR, respectively.

## 4 Discussion

The present study demonstrated that WHR and the combination of BIA parameters with WHR were correlated with ALT level, ALT elevation, and MASLD among youth with overweight and obesity. These relationships were more prominent in the pubertal group. In addition, BIA parameters combined with WHR were stronger predictors than WHR alone. Among the combined parameters, PBF-WHR and VFA-WHR were correlated with the MASLD, even after adjusting for age, sex, and puberty. Moreover, these relationships were significant in youth with normal ALT levels.

In our study, WHR emerged as the sole anthropometric indicator correlated with ALT level in the total population. WHR accounts for both waist and hip measurements, making it a more comprehensive indicator of body fat distribution. The inclusion of hip circumference provides additional information about fat distribution in the lower body, which is relevant because individuals with a higher WHR tend to store more fat in the abdominal area, particularly around the visceral organs, a characteristic feature of individuals at risk for steatotic liver disease (1, 21). Pimenta et al. reported that WHR is strongly correlated with abdominal body fat and body fat distribution (22). A population-based study reported that WHR is a more accurate predictor of severe liver disease than BMI (23). Among anthropometric measurements, including BMI, WC, and WHtR, Zheng et al. reported that WHR was the most powerful predictor of NAFLD in adults (24).

The associations between BIA parameters and pediatric NAFLD have been investigated in several studies. A Japanese study reported that VFA was correlated with NAFLD in children (25). In another Japanese study, magnetic resonance imaging revealed that BIA parameters are associated with total psoas muscle surface area in children with obesity (26). A Chinese study reported that BIA was superior to anthropometric indices in terms of predicting NAFLD among children (27). Based on this evidence, we explored the associations of MASLD with BIA parameters as well as BIA parameters combined with anthropometric measurements. In our study, PBF-WHR and VFA-WHR were associated with MASLD in all subgroups except the prepubertal group, while WHR, SMM/WHR, FMM/WHR, and ASM/WHR were not significantly correlated with MASLD. Therefore, the inclusion of PBF-WHR and VFA-WHR in our study was of significant value for the prediction of MASLD. Choi et al. reported that PBF and the fat mass index are associated with ALT levels, whereas FFM is not (28). The robust relationships between PBF-WHR and VFA-WHR with MASLD can be explained as follows. First, excessive visceral fat accumulation resulting from the infiltration of macrophages among hypertrophied adipocytes is closely associated with the elevation of inflammatory cytokine levels and diminished production of adipokines such as adiponectin (29). A previous study also reported that PBF is closely associated with chronic inflammation in the youth (30). Chronic inflammation plays a crucial role in the development of steatotic liver disease by inducing insulin resistance and promoting the accumulation of lipids in the liver (29). VFA and PBF are linked to insulin resistance and steatotic liver disease via reduced hepatic clearance of insulin (exacerbating hyperinsulinemia), increased production of triglyceride-rich lipoproteins, and elevated hepatic glucose production (31). Notably, examination of the relationship between ALT level elevation and BIA-related parameters revealed that fat-related indicators were significantly associated in boys, while muscle-related indicators were significantly associated in girls. This suggests a potential sex-based difference in the effect of fat and muscle on the occurrence of hepatic fat deposition.

Although ALT is a valuable biomarker, it is not a reliable or accurate predictor of the presence of MASLD (32). Therefore, steatotic liver disease can be underdiagnosed in youth with normal ALT levels, given that the current guidelines recommend imaging studies such as ultrasonography for NAFLD screening, primarily in children with abnormal ALT levels (8). Moreover, a cohort study reported that 14% of patients with NAFLD had normal ALT levels (33). In our study, PBF-WHR and VFA-WHR were correlated with MASLD even in youth with normal ALT levels, whereas WHR alone was not significantly associated with MASLD. These findings suggest that including BIA parameters in the selection criteria for MASLD screening, even in patients with normal ALT levels, could be a beneficial approach for enhancing diagnostic accuracy.

The effectiveness of BIA parameters was notably greater in the pubertal group than in the prepubertal group; our study also revealed significant differences in most BIA parameters between the prepubertal and pubertal groups. This difference can be attributed to the significant changes in body composition, including muscle and fat, that occur during puberty (34). Puberty is a period marked by rapid growth, hormonal changes, and alterations in body composition (35, 36). These changes, including the development of lean muscle mass and the redistribution of fat, can affect the accuracy and relevance of BIA measurements. During puberty, individuals experience substantial growth in muscle mass, which affects BIA-derived measurements (37). Additionally, the redistribution of fat, especially in the abdominal and visceral regions, is characteristic of puberty and influences the parameters related to body fat in BIA. Thus, BIA parameters have become more informative and relevant for assessing metabolic health and risk factors, including steatotic liver disease, in pubertal individuals owing to these dynamic changes in body composition during puberty (36). A longitudinal study reported that the change in energy expenditure during puberty was associated with body composition change (38). The accuracy of BIA in capturing these changes makes it a valuable tool for understanding the associations between body composition and health outcomes during this critical developmental stage.

This study has some limitations. First, our study had a retrospective design and was limited to the Korean population. Second, MASLD was diagnosed using ultrasonography, although biopsy constitutes the gold standard for the diagnosis of steatotic liver disease. Finally, genetic and environmental factors, including nutrition and physical activity, were not considered. However, we propose novel parameters involving the integration of WHR with BIA parameters and show that these parameters are superior to simple anthropometric measurements for the prediction of MASLD even in young patients with normal ALT levels. To the best of our knowledge, this study is the first to experimentally demonstrate the potential of combined parameters using anthropometric data and BIA parameters, indicating the need for more studies with large samples to validate the usefulness of these parameters.

In conclusion, our study demonstrated that BIA parameters combined with WHR exhibited significant correlations with ALT level, ALT elevation, and MASLD and that these relationships were more pronounced in pubertal individuals. The combination of BIA parameters related to fat composition, such as PBF and VFA with WHR, demonstrated stronger associations with MASLD than did WHR alone. Moreover, these relationships were sustained even in youth with normal ALT levels, suggesting the potential of BIA parameters for MASLD screening in such cases. In summary, our study highlights the value of BIA parameters combined with WHR as a promising approach for MASLD screening in youth with overweight and obesity, providing a less burdensome and potentially more effective alternative to traditional methods.

## Data availability statement

The raw data supporting the conclusions of this article will be made available by the authors, without undue reservation.

## Ethics statement

The studies involving humans were approved by the Institutional Review Board of Yonsei University Yongin Severance Hospital. The studies were conducted in accordance with the local legislation and institutional requirements. The requirement for informed consent was waived.

## Author contributions

KS: Conceptualization, Formal analysis, Investigation, Methodology, Writing – original draft. ES: Data curation, Resources, Writing – review & editing. HY: Formal analysis, Methodology, Writing – review & editing. SJ: Methodology, Writing – review & editing. HS: Methodology, Writing – review & editing. HC: Methodology, Writing – review & editing. E-KK: Methodology, Writing – review & editing. Y-JK: Conceptualization, Formal analysis, Investigation, Methodology, Writing – review & editing. J-WL: Supervision, Writing – review & editing.
